# Machine learning prediction models for visual impairment in Chinese adults aged ≥ 45 years with cardiovascular metabolic diseases: a population-based study using CHARLS

**DOI:** 10.1186/s12886-025-04596-6

**Published:** 2025-12-30

**Authors:** Yuhao Liu, Riyan Zhang, Duoduo Xie, Min Liu, Guanshun Yu, Zhong Lin, Jia Qu, Ronghan Wu

**Affiliations:** National Clinical Research Center for Ocular Diseases, Eye Hospital, Wenzhou Medical University, Wenzhou, 325027 China

**Keywords:** Visual impairment, Cardiovascular metabolic diseases, Adults aged 45 years and older, Machine learning–based prediction model

## Abstract

**Background:**

There has been a growing prevalence of cardiovascular metabolic diseases (CMD) in adults aged ≥ 45 years, and vision impairment (VI) is highly prevalent in this population. The objective of this study was to explore the critical determinants of VI in individuals affected by CMD and to develop risk prediction models.

**Methods:**

We analyzed data collected in 2011 (*n* = 1,926) and 2015 (*n* = 3,033) within the China Health and Retirement Longitudinal Study (CHARLS). Risk factors were selected using the least absolute shrinkage and selection operator (LASSO) regression followed by multivariable logistic regression analysis. Eight machine learning (ML) algorithms were applied: LR, GBM, XGBoost, LightGBM, CatBoost, AdaBoost, NN, and SVM. The evaluation of model performance incorporated ROC curves, calibration assessments, and decision curve analysis.

**Results:**

Eleven predictors demonstrated significant links to VI in CMD patients: hearing impairment, depressive symptoms, pain, lower uric acid levels, poorer self-rated health, functional limitations, multimorbidity, reduced cognitive function, poorer sleep quality, and histories of glaucoma and cataract surgery. Among the eight ML algorithms, LR achieved the most stable performance, with AUCs of 0.705 (2015 training set), 0.693 (2015 internal validation set), and 0.695 (2011 temporal validation set). Shapley Additive exPlanations (SHAP) analysis ranked the relative contribution of predictors, and a nomogram was developed for individualized risk estimation.

**Conclusions:**

We established an LR-based prediction model for VI in patients with CMD aged ≥ 45 years, exhibiting stable accuracy and favorable interpretability in clinical settings. This tool may support timely recognition and intervention of eye health risks in CMD patients aged ≥ 45 years, particularly in settings with limited ophthalmic resources.

**Supplementary Information:**

The online version contains supplementary material available at 10.1186/s12886-025-04596-6.

## Introduction

Visual impairment (VI) represents a major public health issue, affecting more than 2.2 billion individuals worldwide and substantially reducing quality of life [1, 2]. Leading causes include correctable disorders, notably uncorrected refractive errors and cataracts, alongside irreversible diseases like age-related macular degeneration and diabetic retinopathy [3]. Beyond its direct impact on vision, VI restricts daily functioning, contributes to psychological distress, increases caregiver burden, and places a considerable strain on healthcare systems [4–6]. Recent evidence from a nationally representative sample of adults in the United States shows that vision-related functional limitations are associated with a higher risk of suicidal ideation, highlighting the substantial psychological and mental health burden of VI [7].

Cardiometabolic diseases (CMD)—comprising hypertension, diabetes, dyslipidemia, coronary artery disease, and stroke—have been consistently associated with elevated risk of VI [8]. Mechanistically, hyperglycemia, hypertension, and lipid abnormalities can damage the retina and optic nerve [8]. Meanwhile, systemic vascular events, such as coronary disease and stroke, impair cerebral and ocular circulation [9, 10]. Given the high burden of CMD patients aged ≥ 45 years and its cumulative effect on ocular microvasculature and systemic metabolism, patients with CMD are increasingly recognized as a high-risk population for VI [11].

Despite this elevated risk, the timely assessment of VI in CMD patients remains challenging. Most risk evaluations still depend on standard ophthalmic or refractive examinations, yet CMD patients typically present to internal medicine rather than ophthalmology, especially in primary care settings. This pattern contributes to delayed vision screening and underdiagnosis. Moreover, there is currently no prediction tool specifically designed to identify VI risk in CMD populations.

Recently, machine learning (ML) models have been widely applied for early risk prediction of chronic conditions such as depression, cognitive decline, and sarcopenia [12–14]. However, no ML-based studies have targeted VI prediction in patients with CMD. To address this gap, we used data from Waves 1 (2011) and 3 (2015) of the China Health and Retirement Longitudinal Study (CHARLS) to develop and validate ML models for predicting VI in Chinese adults aged ≥ 45 years with CMD. By systematically analyzing demographic, clinical, and biochemical features, we identified key predictors and compared multiple ML algorithms. Our goal was to create an accurate and practical tool model for recognizing individuals at elevated risk and guiding timely clinical interventions to prevent vision loss in CMD populations.

## Methods

### Study data and population

CHARLS, a longitudinal cohort survey, was established to reflect the demographic and health profiles of the Chinese middle-aged and elderly population [15]. The baseline investigation, conducted from June 2011 to March 2012, employed a multi-stage stratified probability cluster sampling design across 28 provinces. Follow-up assessments were carried out at intervals of two to three years. Approval for the study was obtained from the Institutional Review Board of Peking University (IRB00001052-11015). Written informed consent was secured from all participants before participation.

We used two CHARLS waves (2011 and 2015), the only survey rounds that included blood-based biomarkers. Data from 2015 were used to construct the prediction model, and the 2011 sample provided temporal validation. Both surveys collected extensive information on demographics, health conditions, functional status, lifestyle behaviors, and biological markers through structured questionnaires and physical examinations.

**Fig. 1:**
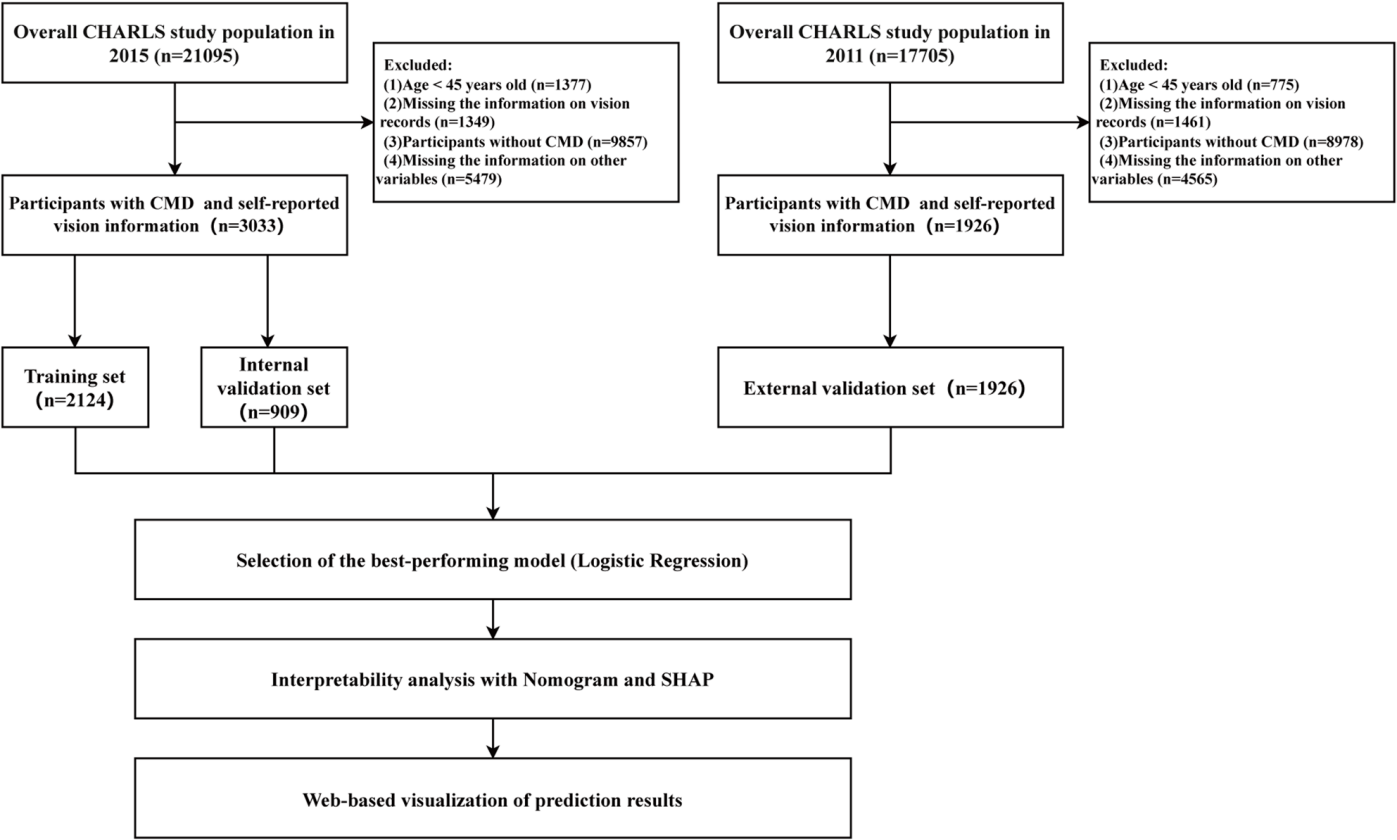
Flowchart of participant selection and study design

Eligible participants were individuals aged ≥ 45 years who met the diagnostic criteria for cardiometabolic disease (CMD). Individuals lacking vision-related questionnaire data or insufficient information to ascertain CMD status were excluded. After applying these criteria, 3,033 participants from the 2015 wave were retained for model development, and 1,926 participants from the 2011 wave constituted the validation cohort. The selection process is summarized in Fig. 1. This study was conducted in accordance with the principles of the Declaration of Helsinki.

### Definitions of CMD and VI

CMD was defined as having at least one condition (e.g., hypertension, dyslipidemia, diabetes, heart disease, or stroke), based on self-report or clinical measurements when available, consistent with previous studies [16, 17]. The definition of VI followed prior CHARLS research [18, 19].

In CHARLS, visual function was assessed using two self-reported items from the CHARLS questionnaire. Participants were asked: (1) “How is your vision for distant tasks (e.g., recognizing someone across the street)?” (2) “How is your vision for near tasks (e.g., reading a newspaper)?” Responses were evaluated using a five-category scale ranging from excellent to poor. Participants who selected “poor” on either item were classified as visually impaired, while those giving other responses to both questions were considered not visually impaired.

### Candidate predictors of VI

Informed by prior research and expert clinical judgment, this study incorporated a comprehensive set of sociodemographic, lifestyle, health-related, comorbidity, and laboratory variables as potential predictors of VI [20–23]. Variable details are available in the Supplementary Material.

### Statistical analysis

All data processing and statistical modeling were performed in R (version 4.4.3). Continuous variables are presented as median values with interquartile ranges, while categorical variables are summarized as counts (n) and percentages (%). Group comparisons were analyzed using statistical methods suitable for the data type, with Mann–Whitney U applied to continuous variables and chi-square or Fisher’s exact to categorical variables; a *p*-value < 0.05 (two-tailed) indicated statistical significance.

### Feature selection

Key predictors were identified using the least absolute shrinkage and selection operator (LASSO) regression. Optimization of the penalty parameter was carried out using ten-fold cross-validation, and the λ1se criterion was chosen to balance model simplicity with predictive performance. Following LASSO selection, variables were re-evaluated in a multivariable logistic regression framework. Predictors with statistical significance at *p* < 0.05 were incorporated into the final predictive model.

### Model development and validation

The 2015 CHARLS dataset was used for model training, with 70% of participants randomly assigned to the training set and 30% to the internal validation set, and the 2011 wave served as a temporal validation cohort to examine model generalizability. The same predictors were used across both datasets to maintain consistency. Eleven predictors identified through feature selection were incorporated into eight ML models to predict VI. The models encompassed logistic regression (LR), support vector machine (SVM), neural networks (NN), and six ensemble approaches: gradient boosting machine (GBM), eXtreme gradient boosting (XGBoost), light gradient boosting machine (LightGBM), categorical boosting (CatBoost), and adaptive boosting (AdaBoost).

Model training involved hyperparameter optimization using grid search, while 10-fold cross-validation was employed to improve generalizability and mitigate overfitting. The tuning ranges and optimal values for all key hyperparameters across the eight ML models are summarized in Supplementary Table S1. Model performance was evaluated with multiple classification indices such as accuracy, precision, and the F1-score. Receiver operating characteristic (ROC) curves were generated, and the area under the curve (AUC) was used to measure model discrimination, with AUC ≥ 0.70 regarded as acceptable discrimination [24]. Calibration was examined by plotting predicted probabilities against observed outcomes, and alignment with the diagonal reference line indicated better agreement. Real-world clinical utility was further analyzed with decision curve analysis (DCA), quantifying net benefit across a range of probability thresholds.

### Model interpretation

To enhance interpretability, we employed the Shapley Additive exPlanations (SHAP) framework, a model-agnostic approach derived from cooperative game theory [25]. SHAP analysis assigns an importance value to every variable, with higher values indicating stronger positive effects on the predicted risk of VI, and lower values reflecting a mitigating influence. This approach allowed us to visualize and interpret the relative importance of the 11 key predictors retained in the final model.

Furthermore, a nomogram was developed using these 11 predictors, offering a practical tool for individualized risk assessment. The nomogram offers a straightforward tool for clinical use, presenting statistical predictions in a visual format to aid decision-making for middle-aged and older adults with CMD.

## Result

### Baseline characteristics

The training cohort included 3,033 individuals with CMD from the 2015 wave of CHARLS, of whom 1,030 (33.9%) self-reported VI. The validation cohort comprised 1,926 CMD participants from the 2011 wave, with 718 (37.3%) reporting VI.

Table 1 summarizes and compares demographic characteristics, clinical history, and laboratory indicators between participants with and without VI. Significant differences (*p* < 0.05) were observed across numerous variables. Specifically, individuals in the VI group tended to be female, from rural regions, have a greater number of chronic conditions, report a history of cataract surgery or glaucoma, and exhibit more frequent depressive symptoms. They also reported lower levels of life satisfaction and anticipated longevity. Additionally, those with VI demonstrated poorer functional status, with more limitations in basic and instrumental daily functioning (ADL/IADL), and higher rates of hearing impairment and pain.

**Table 1:** Baseline characteristics of study participants with cardiovascular metabolic diseases in 2015 (*n* = 3,033)

Variables	Overall	Non-visual impairment	Visual impairment	*p*-value
	(*n* = 3033)	(*n* = 2003)	(*n* = 1030)	
Gender				< 0.001
Male	1517 (50.02%)	1063 (53.07%)	454 (44.08%)	
Female	1516 (49.98%)	940 (46.93%)	576 (55.92%)	
Marital				0.022
Married	2672 (88.10%)	1784 (89.07%)	888 (86.21%)	
Single	361 (11.90%)	219 (10.93%)	142 (13.79%)	
Family residence				< 0.001
Rural	1762 (58.09%)	1113 (55.57%)	649 (63.01%)	
Urban	1271 (41.91%)	890 (44.43%)	381 (36.99%)	
Education level				< 0.001
Less than elementary school	1063 (35.05%)	631 (31.50%)	432 (41.94%)	
Elementary school	793 (26.15%)	544 (27.16%)	249 (24.17%)	
Middle school	731 (24.10%)	492 (24.56%)	239 (23.20%)	
High school or above	446 (14.70%)	336 (16.77%)	110 (10.68%)	
Sleeping time				< 0.001
≤ 6 h	1631 (53.78%)	1036 (51.72%)	595 (57.77%)	
6–8 h	1135 (37.42%)	802 (40.04%)	333 (32.33%)	
> 8 h	267 (8.80%)	165 (8.24%)	102 (9.90%)	
Sleep quality				< 0.001
Very good	1450 (47.81%)	1048 (52.32%)	402 (39.03%)	
Good	445 (14.67%)	307 (15.33%)	138 (13.40%)	
Fair	505 (16.65%)	320 (15.98%)	185 (17.96%)	
Poor	633 (20.87%)	328 (16.38%)	305 (29.61%)	
Smoking Status				0.028
No	1600 (52.75%)	1028 (51.32%)	572 (55.53%)	
Yes	1433 (47.25%)	975 (48.68%)	458 (44.47%)	
Drinking Status				0.003
No	1973 (65.05%)	1266 (63.21%)	707 (68.64%)	
Yes	1060 (34.95%)	737 (36.79%)	323 (31.36%)	
Hypertension				0.607
No	1144 (37.72%)	762 (38.04%)	382 (37.09%)	
Yes	1889 (62.28%)	1241 (61.96%)	648 (62.91%)	
Diabetes				0.257
No	2049 (67.56%)	1367 (68.25%)	682 (66.21%)	
Yes	984 (32.44%)	636 (31.75%)	348 (33.79%)	
Dyslipidemia				0.007
No	1890 (62.31%)	1282 (64.00%)	608 (59.03%)	
Yes	1143 (37.69%)	721 (36.00%)	422 (40.97%)	
Cancer				0.086
No	2972 (97.99%)	1969 (98.30%)	1003 (97.38%)	
Yes	61 (2.01%)	34 (1.70%)	27 (2.62%)	
Lung disease				0.02
No	2517 (82.99%)	1685 (84.12%)	832 (80.78%)	
Yes	516 (17.01%)	318 (15.88%)	198 (19.22%)	
Psych-problem				0.736
No	2969 (97.89%)	1962 (97.95%)	1007 (97.77%)	
Yes	64 (2.11%)	41 (2.05%)	23 (2.23%)	
Asthma				0.003
No	2805 (92.48%)	1873 (93.51%)	932 (90.49%)	
Yes	228 (7.52%)	130 (6.49%)	98 (9.51%)	
Liver disease				0.243
No	2764 (91.13%)	1834 (91.56%)	930 (90.29%)	
Yes	269 (8.87%)	169 (8.44%)	100 (9.71%)	
Heart disease				< 0.001
No	1961 (64.66%)	1338 (66.80%)	623 (60.49%)	
Yes	1072 (35.34%)	665 (33.20%)	407 (39.51%)	
Stroke				< 0.001
No	2843 (93.74%)	1900 (94.86%)	943 (91.55%)	
Yes	190 (6.26%)	103 (5.14%)	87 (8.45%)	
Kidney disease				0.001
No	2615 (86.22%)	1756 (87.67%)	859 (83.40%)	
Yes	418 (13.78%)	247 (12.33%)	171 (16.60%)	
Stomach or other digestive disease				< 0.001
No	2008 (66.21%)	1381 (68.95%)	627 (60.87%)	
Yes	1025 (33.79%)	622 (31.05%)	403 (39.13%)	
Memory-related disease				0.02
No	2935 (96.77%)	1949 (97.30%)	986 (95.73%)	
Yes	98 (3.23%)	54 (2.70%)	44 (4.27%)	
Arthritis or rheumatism				< 0.001
No	1588 (52.36%)	1131 (56.47%)	457 (44.37%)	
Yes	1445 (47.64%)	872 (43.53%)	573 (55.63%)	
ADL				< 0.001
Non-disability	2324 (76.62%)	1626 (81.18%)	698 (67.77%)	
Disability	709 (23.38%)	377 (18.82%)	332 (32.23%)	
IADL				< 0.001
Non-disability	2319 (76.46%)	1640 (81.88%)	679 (65.92%)	
Disability	714 (23.54%)	363 (18.12%)	351 (34.08%)	
Life satisfaction				< 0.001
Not at All Satisfied	43 (1.42%)	17 (0.85%)	26 (2.52%)	
Not Very Satisfied	197 (6.50%)	107 (5.34%)	90 (8.74%)	
Some What Satisfied	1554 (51.24%)	1021 (50.97%)	533 (51.75%)	
Very Satisfied	1050 (34.62%)	721 (36.00%)	329 (31.94%)	
Completely Satisfied	189 (6.23%)	137 (6.84%)	52 (5.05%)	
Health insurance				0.634
No	200 (6.59%)	129 (6.44%)	71 (6.89%)	
Yes	2833 (93.41%)	1874 (93.56%)	959 (93.11%)	
Hearing impairment				< 0.001
No	2581 (85.10%)	1818 (90.76%)	763 (74.08%)	
Yes	452 (14.90%)	185 (9.24%)	267 (25.92%)	
Pain				< 0.001
No	1996 (65.81%)	1451 (72.44%)	545 (52.91%)	
Yes	1037 (34.19%)	552 (27.56%)	485 (47.09%)	
Self-expectations of health status				< 0.001
Almost impossible	508 (16.75%)	267 (13.33%)	241 (23.40%)	
Not very likely	648 (21.36%)	362 (18.07%)	286 (27.77%)	
Maybe	1111 (36.63%)	805 (40.19%)	306 (29.71%)	
Very likely	367 (12.10%)	277 (13.83%)	90 (8.74%)	
Almost certain	399 (13.16%)	292 (14.58%)	107 (10.39%)	
BMI category				0.153
Underweight	122 (4.02%)	78 (3.89%)	44 (4.27%)	
Normal weight	1507 (49.69%)	974 (48.63%)	533 (51.75%)	
Overweight	1154 (38.05%)	791 (39.49%)	363 (35.24%)	
Obese	250 (8.24%)	160 (7.99%)	90 (8.74%)	
Depression				< 0.001
No	1905 (62.81%)	1409 (70.34%)	496 (48.16%)	
Yes	1128 (37.19%)	594 (29.66%)	534 (51.84%)	
Cataract surgery				0.001
No	2948 (97.20%)	1961 (97.90%)	987 (95.83%)	
Yes	85 (2.80%)	42 (2.10%)	43 (4.17%)	
Glaucoma				0.001
No	2989 (98.55%)	1984 (99.05%)	1005 (97.57%)	
Yes	44 (1.45%)	19 (0.95%)	25 (2.43%)	
Age, Median (IQR)	62.00 (55.00–67.00)	62.00 (55.00–67.00)	62.00 (55.00–68.00)	0.379
Chronic conditions, Median (IQR)	3.00 (2.00–4.00)	2.00 (2.00–4.00)	3.00 (2.00–4.00)	< 0.001
Grip, Median (IQR)	30.50 (24.50–38.00)	31.50 (25.50–39.00)	29.00 (23.10–36.00)	< 0.001
Cognition, Median (IQR)	11.50 (9.00-13.50)	12.00 (9.50–14.00)	10.75 (8.00–13.00)	< 0.001
Waist, Median (IQR)	89.00 (81.60–96.20)	89.50 (82.50-96.55)	88.00 (80.40–95.80)	< 0.001
WBC, Median (IQR)	5.85 (4.90-7.00)	5.90 (4.90–7.08)	5.80 (4.80–6.90)	0.203
PLT, Median (IQR)	201.00 (161.00-244.00)	200.00 (162.00-243.00)	203.00 (158.00-248.00)	0.833
HbA1c, Median (IQR)	5.90 (5.60–6.40)	5.90 (5.60–6.40)	5.90 (5.60–6.40)	0.637
HB, Median (IQR)	13.80 (12.80–15.00)	13.90 (12.90–15.10)	13.60 (12.60–14.80)	< 0.001
GLU, Median (IQR)	97.30 (90.09-111.71)	99.10 (90.09-113.51)	97.30 (90.09-111.71)	0.104
TC, Median (IQR)	183.78 (162.55-208.88)	183.78 (162.55-208.88)	182.82 (161.78-208.11)	0.685
TG, Median (IQR)	127.43 (91.15-184.96)	130.09 (91.15-189.38)	123.89 (90.49-179.65)	0.06
HDLC, Median (IQR)	48.65 (41.70-55.98)	48.65 (41.70–55.60)	49.03 (42.08–56.37)	0.223
LDLC, Median (IQR)	101.93 (84.94-120.85)	101.54 (84.94-120.08)	102.70 (84.17-121.91)	0.425
CRP, Median (IQR)	1.60 (0.90–2.90)	1.70 (0.90–2.90)	1.50 (0.80–2.80)	0.053
UA, Median (IQR)	4.90 (4.10–5.90)	5.00 (4.20-6.00)	4.80 (4.00-5.70)	< 0.001
BUN, Median (IQR)	15.13 (12.61–18.21)	15.13 (12.61–18.21)	14.85 (12.61–18.21)	0.66
CR, Median (IQR)	0.77 (0.67–0.91)	0.78 (0.68–0.92)	0.76 (0.65–0.89)	0.001
CysC, Median (IQR)	0.85 (0.74–0.97)	0.85 (0.74–0.96)	0.85 (0.74–0.98)	0.78

Note: Median (interquartile range) was calculated for continuous variables, while frequencies and percentages were determined for categorical variables. The Wilcoxon rank-sum test was used to compare group differences for continuous variables, and Chi-squared tests were employed for categorical variables. ADL means Activities of Daily Living; IADL means Instrumental Activities of Daily Living; BMI means Body Mass Index; WBC means White blood cell; PLT means Platelets; HbA1c means Glycated hemoglobin; HB means Glycated Hemoglobin; GLU means Glucose; TC means Total Cholesterol; TG means Triglycerides; HDLC means High Density Lipoprotein-Cholesterol; LDLC means Low Density Lipoprotein Cholesterol; CRP means C-Reactive Protein. UA means Uric Acid; BUN means Blood Urea Nitrogen; CR means Creatinine; CysC means Cystatin C

Regarding lifestyle and subjective health status, VI participants had lower educational attainment, were less likely to smoke or consume alcohol, had shorter sleep duration, and reported poorer sleep quality. In terms of physical and biochemical measurements, the VI group showed lower grip strength, cognitive scores, hemoglobin levels, uric acid, and serum creatinine, as well as differences in waist circumference compared to the non-VI group. Detailed comparisons are presented in Table 1. Baseline characteristics for the 2011 validation cohort and within-group comparisons in the 2015 training set are provided in Supplementary Tables S2 and S3.

### Feature selection

**Fig. 2:**
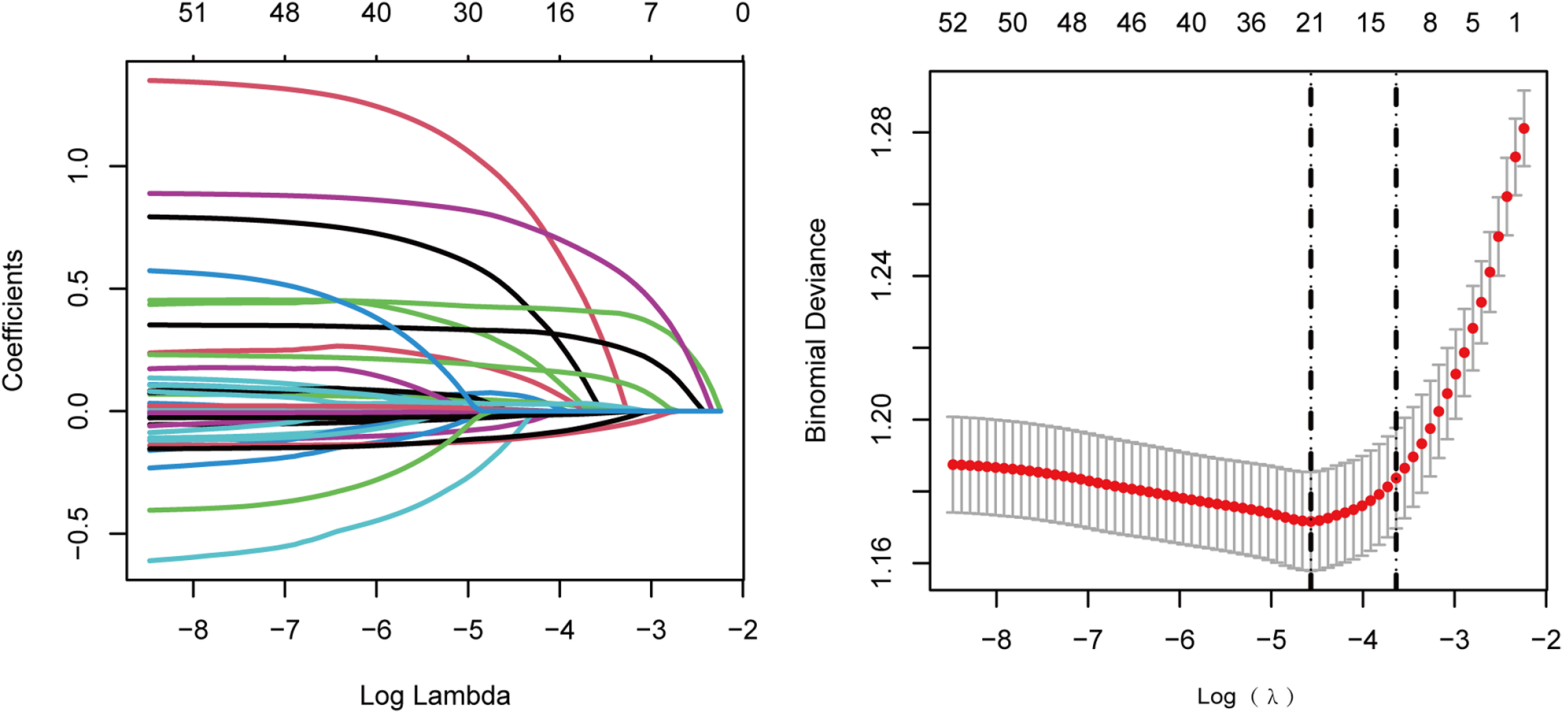
LASSO regression for predictor selection. (**A**) Coefficient profiles across the log(lambda) sequence, with non-zero coefficients retained at the optimal value. (**B**) The optimal lambda was identified by 10-fold cross-validation using the 1-SE rule (right vertical line)

Using LASSO regression followed by multivariable logistic regression, we identified 11 independent predictors of VI in patients aged ≥ 45 years with CMD (Fig. 2; Table 2). These included hearing impairment, depressive symptoms, pain, lower uric acid levels, poorer self-rated health, functional limitations, a greater number of chronic conditions, reduced cognitive scores, poorer sleep quality, and a history of glaucoma or cataract surgery.

**Table 2:** Multivariate logistic regression analysis of the risk factors of visual impairment in middle-aged and older adults with cardiovascular metabolic diseases

Variables	Multivariate analysis OR (95% CI)	*p*-value
Hearing impairment		
No	Reference	
Yes	2.66 (2.14–3.30)	< 0.001
Depression		
No	Reference	
Yes	1.47 (1.21–1.79)	< 0.001
Pain		
No	Reference	
Yes	1.35 (1.12–1.63)	0.002
Self-expectations of health status		
Almost impossible	Reference	
Not very likely	1.00 (0.78–1.29)	0.986
Maybe	0.66 (0.52–0.84)	< 0.001
Very likely	0.58 (0.42–0.80)	< 0.001
Almost certain	0.72 (0.53–0.98)	0.038
IADL		
Non-disability	Reference	
Disability	1.28 (1.05–1.57)	0.013
Sleep quality		
Very good	Reference	
Good	0.94 (0.73–1.20)	0.602
Fair	1.02 (0.80–1.30)	0.855
Poor	1.36 (1.09–1.70)	0.007
Glaucoma		
No	Reference	
Yes	2.01 (1.06–3.82)	0.033
Cataract surgery		
No	Reference	
Yes	1.69 (1.06–2.70)	0.028
Chronic conditions	1.06 (1.01–1.12)	0.016
Cognition	0.97 (0.95–1.00)	0.032
UA	0.90 (0.85–0.96)	< 0.001

Note: IADL means Instrumental Activities of Daily Living; UA means uric acid

### Model development and performance

Eight ML algorithms—LR, SVM, GBM, XGBoost, LightGBM, CatBoost, AdaBoost, and NN—were trained on the 2015 cohort and validated internally as well as temporally in the 2011 cohort.

**Fig. 3:**
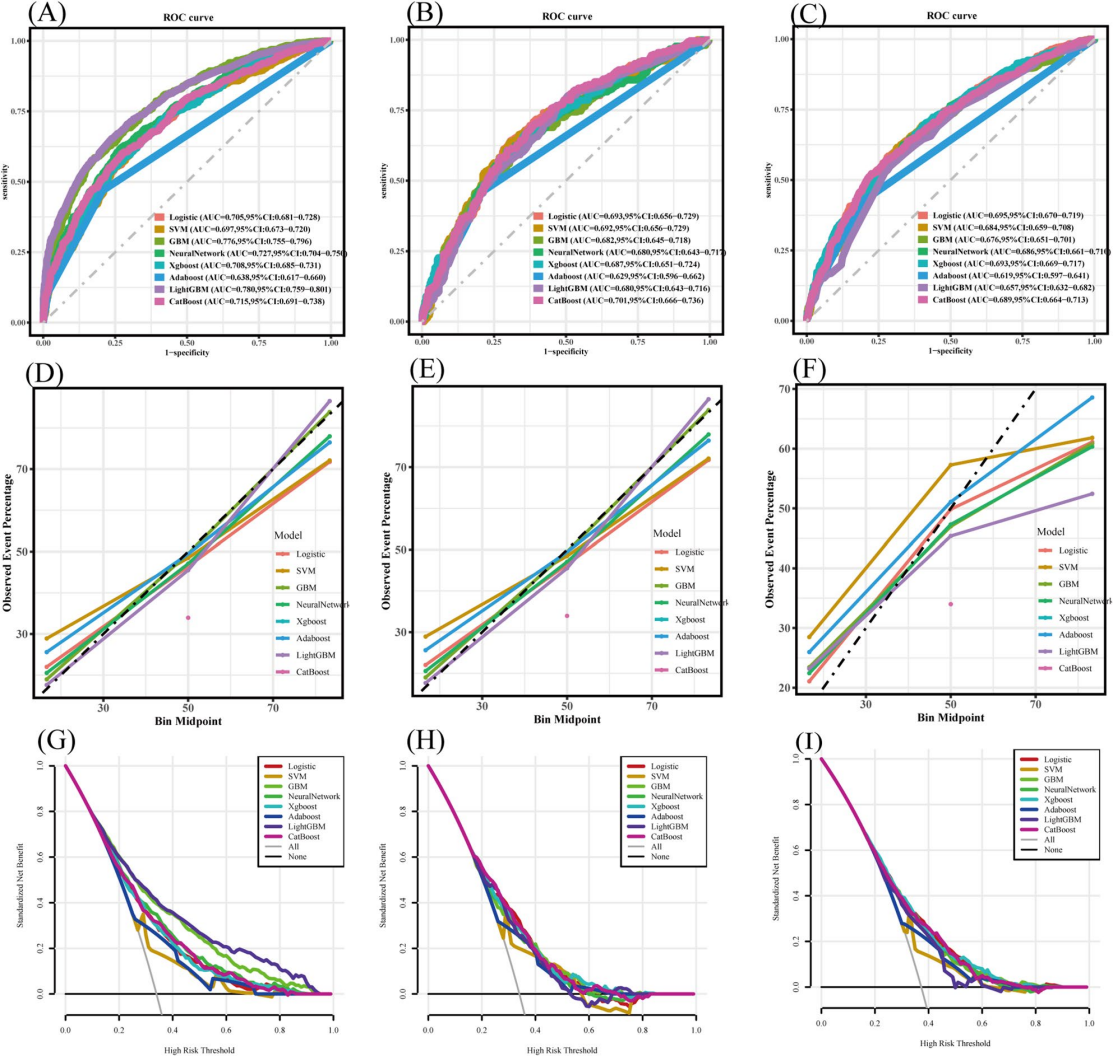
The performance of eight ML models for predicting visual impairment in individuals with cardiovascular metabolic diseases. (**A**) ROC curves for the training set; (**B**) ROC curves for the validation set; (**C**) Calibration curves for the training set; (**D**) Calibration curves for the validation set; (**E**) Decision curve analysis for the training set; (**F**) Decision curve analysis for the validation set

In the training dataset, AUC values ranged from 0.638 (AdaBoost) to 0.780 (LightGBM). Ensemble methods (LightGBM, GBM) and the neural network achieved the highest discrimination, whereas LR reached an AUC of 0.705. In contrast, performance differences diminished in validation: AUCs ranged between 0.629 and 0.701, with LR (0.693–0.695) showing stable discrimination and AdaBoost consistently performing worst across all datasets (Fig. 3).

Calibration analysis indicated that most models, particularly LightGBM, GBM, CatBoost, and LR, showed robust correspondence between predicted and actual risks in the training cohort. DCA further demonstrated that CatBoost, LightGBM, and LR provided greater net clinical benefit than “treat all” or “treat none” strategies across a wide range of thresholds, with comparable stability in validation cohorts.

Performance metrics summarized in Table 3 highlighted the strengths and limitations of each algorithm. LightGBM achieved the highest training accuracy (0.703), while GBM yielded the best specificity (0.737) and precision (0.611). However, in both validation settings, LR yielded the most balanced and consistent results, with accuracy (0.663), sensitivity (0.631), specificity (0.681), and F1-score (0.577).

**Table 3:** Comparison of the performance of predictive models for visual impairment in middle-aged and older adults with cardiovascular metabolic diseases

Model	Threshold	Accuracy	Sensitivity	Specificity	Precision	F1
Training set						
LR	0.327	0.661	0.638	0.673	0.501	0.561
SVM	0.287	0.648	0.693	0.625	0.487	0.572
GBM	0.398	0.742	0.585	0.822	0.628	0.606
NN	0.357	0.694	0.616	0.734	0.543	0.577
Xgboost	0.498	0.633	0.716	0.591	0.473	0.570
Adaboost	0.181	0.683	0.469	0.793	0.538	0.501
LightGBM	0.367	0.725	0.653	0.762	0.585	0.617
CatBoost	0.586	0.684	0.599	0.728	0.531	0.563
Internal validation set						
LR	0.336	0.675	0.641	0.693	0.518	0.573
SVM	0.290	0.674	0.638	0.693	0.517	0.571
GBM	0.308	0.645	0.657	0.638	0.483	0.557
NN	0.348	0.672	0.605	0.707	0.515	0.557
Xgboost	0.498	0.650	0.667	0.642	0.489	0.564
Adaboost	0.180	0.680	0.460	0.793	0.534	0.494
LightGBM	0.246	0.612	0.767	0.532	0.458	0.573
CatBoost	0.581	0.645	0.693	0.620	0.484	0.570
External validation set						
LR	0.355	0.668	0.584	0.718	0.551	0.567
SVM	0.290	0.659	0.616	0.685	0.538	0.574
GBM	0.366	0.660	0.542	0.730	0.544	0.543
NN	0.337	0.650	0.624	0.666	0.526	0.571
Xgboost	0.498	0.631	0.698	0.591	0.504	0.585
Adaboost	0.180	0.661	0.428	0.800	0.559	0.485
LightGBM	0.389	0.650	0.549	0.710	0.530	0.539
CatBoost	0.587	0.681	0.522	0.775	0.580	0.549

Note: LR means Logistic Regression; SVM means Support Vector Machine; GBM means Gradient Boosting Machine; NN means Neural Network; Xgboost means eXtreme Gradient Boosting; AdaBoost means Adaptive Boosting; LightGBM means Light Gradient Boosting Machine; Catboost means Categorical Boosting. Accuracy denotes the proportion of correctly predicted samples among the total samples. Sensitivity reflects the model’s ability to identify actual positive cases correctly. Specificity indicates the model’s ability to identify actual negative cases accurately. Precision denotes the fraction of correctly identified positive cases among all instances predicted to be positive. F1 Score represents the harmonic mean of precision and recall, indicating a balanced measure of a model’s ability to identify both positive and negative cases accurately

Taken together, LR outperformed more complex models in terms of robustness, calibration, clinical utility, and generalizability, supporting its selection as the preferred model for implementation.

### Model interpretation and visualization

**Fig. 4:**
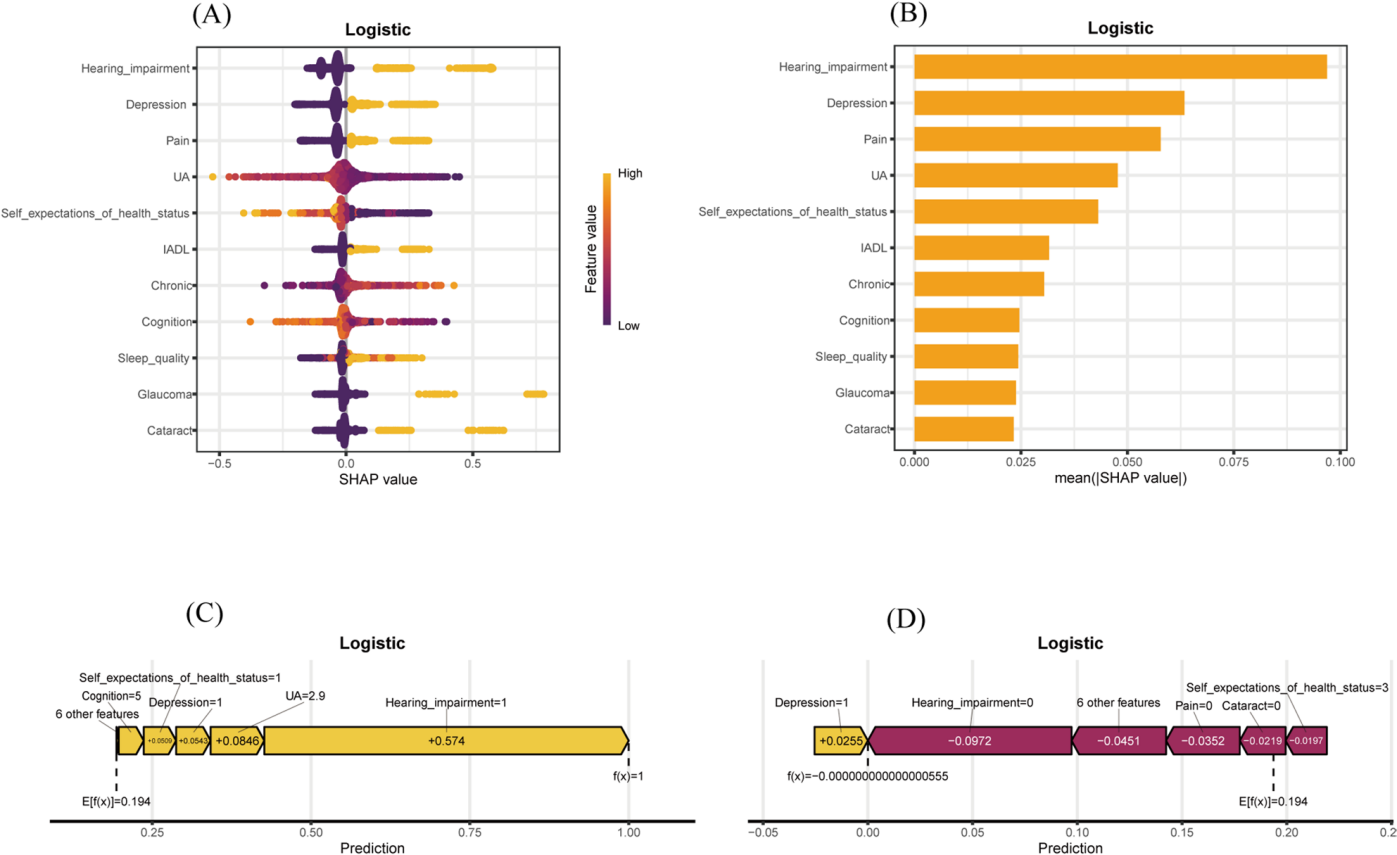
SHAP interpretation of the logistic regression model for VI prediction in individuals with cardiovascular metabolic diseases. (**A**) Bar plot ranking features by mean SHAP values to indicate importance. (**B**) SHAP summary plot displaying the effect and distribution of each variable. (**C**) Force plot of a VI case, illustrating features driving higher risk. (**D**) Force plot of a non-VI case, showing features lowering predicted risk

The logistic regression model was further interpreted using SHAP values. (Fig. 4). The summary plot (Fig. 4A) highlighted hearing impairment, depressive symptoms, pain, lower uric acid, and poorer self-expected health status as the most influential predictors, as reflected by their wide SHAP value distributions and strong contributions to the model.

The ranking based on mean SHAP values (Fig. 4B) confirmed hearing impairment as the leading predictor, followed by depression, pain, uric acid concentration, and self-expected health status. Moderate but notable contributions were observed for IADL limitations, the number of chronic conditions, cognitive scores, sleep quality, and prior glaucoma or cataract surgery.

To further illustrate case-level predictions, SHAP force plots were generated. Figure 4C shows an example of an individual with a high predicted risk of VI, while Fig. 4D represents a case with a low predicted risk. These visualizations offer intuitive insight into how specific features impact individual predictions, thereby enhancing the model’s clinical interpretability.

### Construction of a risk nomogram

**Fig. 5:**
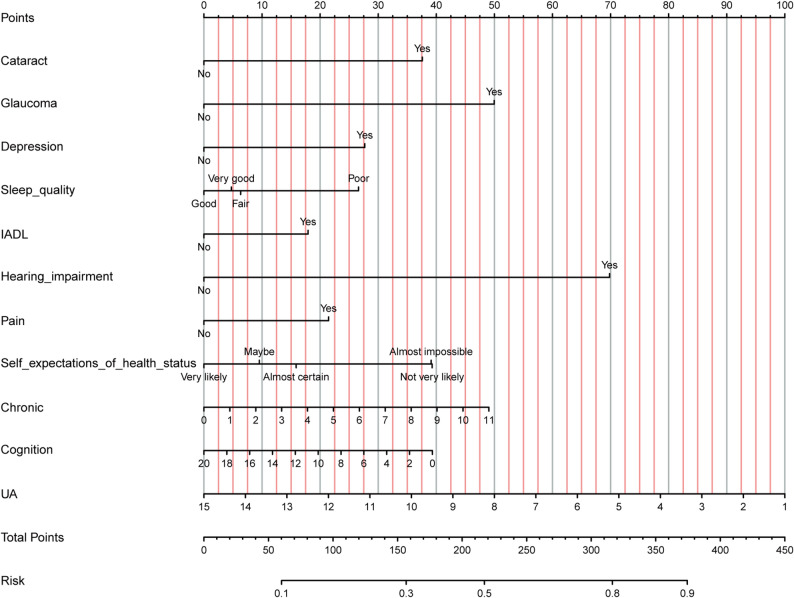
Nomogram for predicting VI risk in individuals with cardiovascular metabolic diseases

Given the stable performance of the LR model across the training and validation cohorts, a risk nomogram was constructed incorporating the 11 independent predictors (Fig. 5). Each variable is displayed along a scaled axis, with corresponding point values assigned. For an individual patient, the sum of these points yields a total score, which can be converted into a predicted probability of VI. Higher total scores correspond to a greater predicted risk of VI among adults aged ≥ 45 with CMD. This nomogram serves as a simple and practical tool for individualized VI risk assessment and may facilitate timely referral to ophthalmologists in primary care settings.

## Discussion

In our study, we utilized cross-sectional datasets from the 2011 and 2015 waves of the CHARLS to investigate the burden of VI in patients with CMD aged ≥ 45 years and to explore factors associated with VI. Our findings revealed that the probability of VI among CMD patients was 37.3% (718/1926) in the 2011 wave and 33.9% (1030/3033) in the 2015 wave. From an initial set of 52 candidate variables, eleven key predictors of VI were identified through LASSO selection and subsequently multivariable logistic regression. These features included hearing impairment, depressive symptoms, pain, serum uric acid levels, self-expected health status, IADL limitations, number of chronic conditions, cognitive function score, sleep quality, glaucoma history, and cataract surgery history. Taken together, these predictors delineate a multidimensional risk profile—spanning sensory, psychological, functional, systemic, and ocular domains—that can be used to stratify CMD patients according to their likelihood of VI and to inform tailored surveillance and management strategies.

Incorporating these variables, we constructed a nomogram to estimate VI risk in this population. This tool offers a practical means of early identification of high-risk individuals, with the potential to facilitate timely clinical decision-making and reduce the burden of irreversible ocular damage over the long term. We further developed and compared eight ML models for the risk prediction of VI. Although GBM and LightGBM achieved the highest AUCs in the training set, both showed notable performance declines in validation, indicating overfitting. By contrast, the LR model maintained stable performance across all datasets, with AUCs around 0.70 and consistent accuracy and F1 scores, thereby demonstrating superior robustness and clinical applicability in detecting VI risk in CMD patients aged 45 ≥ years.

Among the identified predictors, hearing impairments emerged as one of the strongest correlates of VI. This is in line with previous reports indicating that hearing loss and visual impairment frequently coexist among older adults, a phenomenon referred to as dual sensory impairment [26]. This may be explained by the fact that both hearing and visual impairments are hallmarks of biological aging, with individuals relying more on visual input when hearing function declines, thereby exacerbating the burden on the visual system [27]. Clinically, CMD patients with documented hearing loss could therefore be prioritized for more frequent vision assessments, a lower threshold for ophthalmic referral, and counselling about dual sensory impairment, as they may experience greater functional impact from even mild VI. Depression or a pessimistic expectation of future health status may place the body in a state of sustained stress. Such prolonged psychological stress—manifested as worry, anxiety, or fear—can induce long-term inflammation, imbalance of the hypothalamic–pituitary–adrenal (HPA) axis, and metabolic disturbances [28]. These mechanisms may accelerate vascular sclerosis and retinal microvascular damage, thereby increasing the risk of VI [29]. Our results suggest that routine assessment of depressive symptoms and self-expected health status in CMD clinics could help to identify patients at elevated risk who may warrant earlier or more frequent ophthalmic evaluation and counselling regarding VI; in parallel, integrating brief mental health screening and establishing referral pathways to psychological services represent concrete opportunities for personalized prevention.

In individuals with CMD, the presence of chronic pain can substantially limit IADL and impair self-management abilities [30, 31]. These functional limitations may reduce adherence to regular ophthalmic examinations and follow-up care, which in turn may elevate the risk of developing VI. Poor sleep quality, often accompanied by heightened systemic inflammation and metabolic dysregulation, may further exacerbate retinal damage and accelerate visual function decline [32, 33]. Moreover, sleep disorders themselves have been identified as independent predictors of diabetic retinopathy, a major cause of visual decline in adults aged ≥ 45 years [34] From a clinical standpoint, CMD patients reporting chronic pain, IADL disability, or poor sleep quality may benefit from tailored pain management, rehabilitation to maintain independence, evaluation and treatment of sleep disorders, and more intensive ophthalmic surveillance. Incorporating these domains into VI risk assessment could support more individualized follow-up schedules and multidisciplinary interventions.

The coexistence of multiple chronic diseases has been shown to increase vulnerability to VI, since overlapping pathways such as systemic vascular injury and neurodegenerative alterations may jointly contribute to accelerated retinal deterioration and vision loss [35]. In practice, a simple count of chronic conditions—readily available in health records—can be combined with our nomogram to flag multimorbid CMD patients for prioritized ophthalmic referral and closer monitoring. Interestingly, our findings that moderate uric acid levels were associated with a lower prevalence of VI, are consistent with prior evidence in glaucoma, possibly due to the antioxidant capacity of uric acid and its putative role in neuroprotection within the retina [36]. However, these cross-sectional associations do not justify modifying uric acid levels solely to prevent VI; instead, they highlight the potential value of metabolic and oxidative stress markers in refining risk stratification and in identifying subgroups who may be particularly susceptible to visual decline. Finally, both prior cataract surgery and a history of glaucoma were linked to elevated VI risk in CMD populations. This finding aligns with the Global Burden of Disease (GBD) study, which underscores cataract as the foremost cause of blindness globally and highlights glaucoma as the leading cause of irreversible blindness, responsible for an estimated 4.14 million blind cases in 2020—equivalent to 1.4% of the 295 million individuals living with vision loss [37, 38]. From a practical perspective, CMD patients with a history of cataract surgery or glaucoma should be regarded as a particularly vulnerable subgroup. Ensuring regular ophthalmic follow-up, optimizing refractive correction, and achieving good intraocular pressure control in these patients may help to mitigate their residual risk of VI.

This study has several limitations. First, both VI and CMD were identified by self-report rather than clinical confirmation, which may introduce reporting bias. Second, the cross-sectional design precludes causal inference. All reported relationships should therefore be interpreted as associations rather than evidence of direct causation. Third, as CHARLS includes only Chinese participants, the applicability of the model to different populations is unclear. Finally, some potentially important predictors (e.g., disease duration, genetic risk factors, or detailed ophthalmic examinations) were not available and thus could not be incorporated. Future work should validate this model in other large-scale cohorts and integrate additional risk factors. Despite these limitations, our study provides important advances. To our knowledge, this is the first nomogram specifically developed for assessing VI risk in Chinese CMD individuals aged ≥ 45 years. By combining 11 easily obtainable risk factors, the model demonstrated good discrimination in identifying high-risk individuals. Such a tool may support clinicians in timely referral of CMD patients for ophthalmic evaluation and has potential applicability in resource-limited settings.

## Conclusion

In this study, eight ML algorithms were applied to develop a predictive model for VI in patients aged ≥ 45 years with cardiovascular metabolic diseases. Using cross-sectional data from two CHARLS waves, comparative analyses showed that LR achieved the best performance in terms of predictive accuracy, stability, and clinical interpretability. Eleven key predictors were ultimately identified: hearing impairment, depression, chronic pain, lower uric acid levels, negative self-expected health status, IADL disability, multimorbidity, reduced cognitive function, poor sleep quality, cataract surgery history, and glaucoma history. These variables do not imply causality but define a clinically recognizable high-risk profile that can be used to stratify CMD patients according to their probability of VI. The proposed model provides clinicians managing CMD with a simple tool to screen patients vulnerable to VI and to facilitate timely ophthalmic referral.

## Supplementary Information

Below is the link to the electronic supplementary material.

Supplementary Material 1

## Data Availability

All data used in this study are publicly accessible from the CHARLS project website ([http://charls.pku.edu.cn/]).

## Abbreviations

VI: Visual impairment
CHARLS: China Health and Retirement Longitudinal Study
CMD: Cardiovascular Metabolic Diseases
HbA1c: Glycated hemoglobin
ADL: Activities of daily living
IADL: Instrumental activities of daily living
BMI: Body mass index
WBC: White blood cell count
PLT: Platelet count
HDL-C: High-density lipoprotein cholesterol
LDL-C: Low-density lipoprotein cholesterol
UA: Uric acid
BUN: Blood urea nitrogen
CRP: C-reactive protein
LASSO: Least absolute shrinkage and selection operator
LR: Logistic regression
GBM: Gradient boosting machine
XGBoost: eXtreme gradient boosting
LightGBM: Light Gradient Boosting Machine
CatBoost: Categorical boosting
AdaBoost: Adaptive boosting
NN: Neural network
SVM: Support vector machine
ROC: Receiver operating characteristic
AUC: Area under the ROC curve
DCA: Decision curve analysis
SHAP: SHapley Additive exPlanations
HPA: Hypothalamic–pituitary–adrenal
GBD: Global Burden of Disease

## References

[1] Vision impairment and blindness. https://www.who.int/news-room/fact-sheets/detail/blindness-and-visual-impairment. Accessed 17 July 2025.

[2] Steinmetz JD, Bourne RRA, Briant PS, Flaxman SR, Taylor HRB, Jonas JB, et al. Causes of blindness and vision impairment in 2020 and trends over 30 years, and prevalence of avoidable blindness in relation to VISION 2020: the right to sight: an analysis for the global burden of disease study. Lancet Global Health. 2021;9:e144–60. 10.1016/S2214-109X(20)30489-7. 33275949 10.1016/S2214-109X(20)30489-7 PMC7820391

[3] World Health Organization (WHO). https://www.who.int. Accessed 17 July 2025.

[4] Yaroshevich EA, Gnezdilova AD. The effect of various visual impairments on the daily activities of older patients. Adv Gerontol (*Uspekhi gerontologii*). 2024;37. 39139115

[5] H C, H X, Z X. A study on the mechanism of how sensory impairment affects depression in the elderly: the mediating roles of daily activity capability and social participation. Front Psychol. 2024;15. 10.3389/fpsyg.2024.1410422. 10.3389/fpsyg.2024.1410422 PMC11578716 39575334

[6] Marques AP, Ramke J, Cairns J, Butt T, Zhang JH, Muirhead D, et al. Global economic productivity losses from vision impairment and blindness. EClinicalMedicine. 2021;35:100852. 10.1016/j.eclinm.2021.100852. 33997744 10.1016/j.eclinm.2021.100852 PMC8093883

[7] Cui Y. Functional limitations and suicidal ideation: Independent, interactive, and cumulative associations in a nationally representative sample. Gen Hosp Psychiatry. 2025;95:148–57. 10.1016/j.genhosppsych.2025.05.006. 40382814 10.1016/j.genhosppsych.2025.05.006

[8] Hj K, Sj C, Hn EY. Chronic disease interventions for people with visual impairment: A systematic review. Appl Nurs Research: ANR. 2021. 10.1016/j.apnr.2021.151446. 60. 10.1016/j.apnr.2021.151446 34247790

[9] Rowe FJ, Hepworth LR, Howard C, Hanna KL, Currie J. Impact of visual impairment following stroke (IVIS study): a prospective clinical profile of central and peripheral visual deficits, eye movement abnormalities and visual perceptual deficits. Disabil Rehabil. 2022;44:3139–53. 10.1080/09638288.2020.1859631. 33347793 10.1080/09638288.2020.1859631

[10] Chatziralli IP, Kazantzis D, Chatzirallis AP, Machairoudia G, Papageorgiou EG, Theodossiadis GP, et al. Cardiometabolic factors and risk of non-arteritic anterior ischemic optic neuropathy: a systematic review and meta-analysis. Graefes Arch Clin Exp Ophthalmol. 2022;260:1445–56. 10.1007/s00417-021-05522-4. 35067769 10.1007/s00417-021-05522-4

[11] GBD 2019 Diseases and Injuries Collaborators. Global burden of 369 diseases and injuries in 204 countries and territories, 1990–2019: a systematic analysis for the global burden of disease study 2019. Lancet. 2020;396:1204–22. 10.1016/S0140-6736(20)30925-9. 33069326 10.1016/S0140-6736(20)30925-9 PMC7567026

[12] Xiao Y, Zhao Z, Su C, Li J, Liu J. An interpretable machine learning model for predicting depression in middle-aged and elderly cancer patients in china: a study based on the CHARLS cohort. BMC Psychiatry. 2025;25:610. 10.1186/s12888-025-07074-x. 40597989 10.1186/s12888-025-07074-x PMC12210965

[13] Wu X, Yao X, Shi J, Tang M, Zhou Q, Chen K. Development and validation of a machine learning model for early screening of high-risk mild cognitive impairment from the multi-cohort data. Int J Med Inf. 2025;203:106030. 10.1016/j.ijmedinf.2025.106030. 10.1016/j.ijmedinf.2025.106030 40633300

[14] Yu P, Zhang X, Sun G, Zeng P, Zheng C, Wang K. Sarcopenia prediction model based on machine learning and SHAP values for community-based older adults with cardiovascular disease in China. Front Public Health. 2025;13:1527304. 10.3389/fpubh.2025.1527304. 40469611 10.3389/fpubh.2025.1527304 PMC12133505

[15] Zhao Y, Hu Y, Smith JP, Strauss J, Yang G. Cohort profile: the China health and retirement longitudinal study (CHARLS). Int J Epidemiol. 2014;43:61–8. 10.1093/ije/dys203. 23243115 10.1093/ije/dys203 PMC3937970

[16] Zhang G, Dong S, Wang L. Construction of a machine learning-based risk prediction model for depression in middle-aged and elderly patients with cardiovascular metabolic diseases in china: a longitudinal study. BMC Public Health. 2025;25:1904. 10.1186/s12889-025-23075-7. 40410764 10.1186/s12889-025-23075-7 PMC12101033

[17] Guo D, Wang Y, Zhao Y, Ding R, Luo Y, Dai W, et al. The longitudinal association between multiple cardiometabolic diseases, socioeconomic status, and depressive symptoms in China. Sci Rep. 2025;15:2971. 10.1038/s41598-025-87516-4. 39849047 10.1038/s41598-025-87516-4 PMC11758380

[18] Determinants of Visual Impairment Among Chinese Middle-Aged and Older Adults. Risk Prediction Model Using Machine Learning Algorithms - PubMed. https://pubmed.ncbi.nlm.nih.gov/39382570/. Accessed 27 May 2025. 10.2196/59810 PMC11481821 39382570

[19] Residential greenness. air pollution and visual impairment: a prospective cohort study - PubMed. https://pubmed.ncbi.nlm.nih.gov/39614249/. Accessed 27 May 2025. 10.1186/s12889-024-20853-7 PMC11606143 39614249

[20] Sun M, Bo Q, Lu B, Sun X, Zhou M. The association of sleep duration with vision impairment in middle-aged and elderly adults: evidence from the China Health and Retirement Longitudinal Study. Front Med. 2021;8. 10.3389/fmed.2021.778117. 10.3389/fmed.2021.778117 PMC8738086 35004745

[21] Gu Y, Cheng H, Liu X, Dong X, Congdon N, Ma X. Prevalence of self-reported chronic conditions and poor health among older adults with and without vision impairment in China: a nationally representative cross-sectional survey. BMJ Open Ophth. 2023;8. 10.1136/bmjophth-2022-001211. 10.1136/bmjophth-2022-001211 PMC9980335 37278435

[22] Associations of vision. impairment and eye diseases with frailty in community-dwelling older adults: a nationwide longitudinal study in China. Br J Ophthalmol. 2024;108(2):310‑316. https://bjo.bmj.com/content/108/2/310 . Accessed 28 May 2025. 10.1136/bjo-2022-322048 36535748

[23] Associations of Vision Impairment and Eye Diseases With Memory Decline Over 4 Years in China and the United States. - ScienceDirect. https://www.sciencedirect.com/science/article/abs/pii/S0002939421001343?via%3Dihub. Accessed 28 May 2025. 10.1016/j.ajo.2021.03.021 33773983

[24] Mandrekar JN. Receiver operating characteristic curve in diagnostic test assessment. J Thorac Oncol. 2010;5:1315–6. 10.1097/JTO.0b013e3181ec173d. 20736804 10.1097/JTO.0b013e3181ec173d

[25] Lundberg S, Lee S-I. A unified approach to interpreting model predictions. 2017. 10.48550/arXiv.1705.07874.

[26] Yeo BSY, Gao EY, Tan BKJ, Ong BDC, Cho RWY, Lim CY, et al. Dual sensory impairment: global prevalence, future projections, and its association with cognitive decline. Alzheimers Dement. 2025;21:e14465. 10.1002/alz.14465. 39887563 10.1002/alz.14465 PMC11851313

[27] Bottari D, Nava E, Ley P, Pavani F. Enhanced reactivity to visual stimuli in deaf individuals. Restor Neurol Neurosci. 2010;28:167–79. 10.3233/RNN-2010-0502. 20404406 10.3233/RNN-2010-0502

[28] Lei AA, Phang VWX, Lee YZ, Kow ASF, Tham CL, Ho Y-C, et al. Chronic Stress-Associated depressive disorders: the impact of HPA axis dysregulation and neuroinflammation on the Hippocampus-A mini review. Int J Mol Sci. 2025;26:2940. 10.3390/ijms26072940. 40243556 10.3390/ijms26072940 PMC11988747

[29] Sabel BA, Wang J, Cárdenas-Morales L, Faiq M, Heim C. Mental stress as consequence and cause of vision loss: the dawn of psychosomatic ophthalmology for preventive and personalized medicine. EPMA J. 2018;9:133–60. 10.1007/s13167-018-0136-8. 29896314 10.1007/s13167-018-0136-8 PMC5972137

[30] Chu J, Weng L, Jin W, Yin X, Xu Q, Xu Z. Pain status and disability in activities of daily living among older adults in china: evidence from CHARLS 2020. Pain Res Manag. 2025;2025:4974163. 10.1155/prm/4974163. 40703499 10.1155/prm/4974163 PMC12286672

[31] Balicki P, Sołtysik BK, Borowiak E, Kostka T, Kostka J. Activities of daily living limitations in relation to the presence of pain in community-dwelling older adults. Sci Rep. 2025;15:15027. 10.1038/s41598-025-00241-w. 40301354 10.1038/s41598-025-00241-w PMC12041235

[32] Irwin MR, Olmstead R, Carroll JE, Sleep Disturbance. Sleep Duration, and inflammation: A systematic review and Meta-Analysis of cohort studies and experimental sleep deprivation. Biol Psychiatry. 2016;80:40–52. 10.1016/j.biopsych.2015.05.014. 26140821 10.1016/j.biopsych.2015.05.014 PMC4666828

[33] Garbarino S, Lanteri P, Bragazzi NL, Magnavita N, Scoditti E. Role of sleep deprivation in immune-related disease risk and outcomes. Commun Biol. 2021;4:1304. 10.1038/s42003-021-02825-4. 34795404 10.1038/s42003-021-02825-4 PMC8602722

[34] Zheng Z, Wang C, Li C, Wu Q, Chen X, Chen H, et al. Meta-Analysis of relationship of sleep quality and duration with risk of diabetic retinopathy. Front Endocrinol (Lausanne). 2022;13:922886. 10.3389/fendo.2022.922886. 35813644 10.3389/fendo.2022.922886 PMC9256993

[35] Zheng DD, Christ SL, Lam BL, Feaster DJ, McCollister K, Lee DJ. Patterns of chronic conditions and their association with visual impairment and health care use. JAMA Ophthalmol. 2020;138:387–94. 10.1001/jamaophthalmol.2020.0052. 32105300 10.1001/jamaophthalmol.2020.0052 PMC7047855

[36] Li S, Shao M, Li D, Tang B, Cao W, Sun X. Association of serum uric acid levels with primary open-angle glaucoma: a 5-year case-control study. Acta Ophthalmol. 2019;97:e356–63. 10.1111/aos.13789. 29673085 10.1111/aos.13789

[37] Vision Loss Expert Group of the Global Burden of Disease Study, GBD 2019 Blindness and Vision Impairment Collaborators. Global estimates on the number of people blind or visually impaired by cataract: a meta-analysis from 2000 to 2020. Eye (Lond). 2024;38:2156–72. 10.1038/s41433-024-02961-1. 10.1038/s41433-024-02961-1 PMC11269584 38461217

[38] Vision Loss Expert Group of the Global Burden of Disease Study, GBD 2019 Blindness and Vision Impairment Collaborators. Global estimates on the number of people blind or visually impaired by glaucoma: A meta-analysis from 2000 to 2020. Eye (Lond). 2024;38:2036–46. 10.1038/s41433-024-02995-5. 38565601 10.1038/s41433-024-02995-5 PMC11269708

