# Supplementary material for: Machine learning prediction models for visual impairment in Chinese adults aged ≥ 45 years with cardiovascular metabolic diseases: a population-based study using CHARLS
Source: BMC Ophthalmol. 2025 Dec 30;26:58. doi: 10.1186/s12886-025-04596-6 (PMC12860091; doi:10.1186/s12886-025-04596-6)
Supplement: Supplementary file 1 — Supplementary Material 1 [file 12886_2025_4596_MOESM1_ESM.docx]

**Supplementary Material**

**Machine Learning Prediction Models for Visual Impairment in Chinese Adults Aged ≥45 Years with Cardiovascular Metabolic Diseases: A Population-Based Study Using CHARLS**

**Supplementary Tables**

**Table S1: Summary of hyperparameter optimization for all machine learning models**

| Model | Hyperparameter | Tuning scope | Optimal hyperparameter |
| --- | --- | --- | --- |
| GBM | n.trees | (100, 110) | 110 |
|  | interaction.depth | (4, 5) | 4 |
|  | shrinkage | (0.05, 0.1) | 0.95 |
| SVM | sigma | (0.01,0.001) | 0.001 |
|  | C | (0.05,0.1) | 0.09 |
| NN | size | (3, 6) | 4 |
|  | decay | (0.3, 0.6) | 0.4 |
| Catboost | iterations | (100, 110) | 100 |
|  | border_count | (10, 20) | 20 |
|  | depth | (1, 4) | 3 |
|  | learning_rate | (0.01, 0.03) | 0.03 |
| Xgboost | nrounds | (5, 10) | 10 |
|  | max_depth | (2, 3) | 3 |
|  | eta | (0.01, 0.001) | 0.001 |
|  | gamma | (0.1, 0.5) | 0.5 |
| Adaboost | mfinal | (1, 2) | 2 |
|  | maxdepth | (2, 3) | 2 |
| LightGBM | nrounds | (1, 5) | 3 |
|  | learning_rate | (0.1, 1) | 1 |
|  | num_threads | (1, 10) | 2 |

**Table S2 Baseline characteristics of study participants in 2011 (n = 1,926).**

| **Variables** | **Overall** | **Non-visual impairment** | **Visual impairment** | ***p-value*** |
| --- | --- | --- | --- | --- |
|  | (n =1926) | (n =1208) | (n =718) |  |
| Gender |  |  |  | <0.001 |
| Male | 931 (48.34%) | 619 (51.24%) | 312 (43.45%) |  |
| Female | 995 (51.66%) | 589 (48.76%) | 406 (56.55%) |  |
| Marital |  |  |  | 0.133 |
| Married | 1686 (87.54%) | 1068 (88.41%) | 618 (86.07%) |  |
| Single | 240 (12.46%) | 140 (11.59%) | 100 (13.93%) |  |
| Family residence |  |  |  | 0.002 |
| Rural | 1133 (58.83%) | 678 (56.13%) | 455 (63.37%) |  |
| Urban | 793 (41.17%) | 530 (43.87%) | 263 (36.63%) |  |
| Education level |  |  |  | <0.001 |
| Less than elementary school | 827 (42.94%) | 449 (37.17%) | 378 (52.65%) |  |
| Elementary school | 463 (24.04%) | 295 (24.42%) | 168 (23.40%) |  |
| Middle school | 410 (21.29%) | 298 (24.67%) | 112 (15.60%) |  |
| High school or above | 226 (11.73%) | 166 (13.74%) | 60 (8.36%) |  |
| Sleeping time |  |  |  | 0.002 |
| ≤6h | 1027 (53.32%) | 616 (50.99%) | 411 (57.24%) |  |
| 6–8h | 761 (39.51%) | 513 (42.47%) | 248 (34.54%) |  |
| >8h | 138 (7.17%) | 79 (6.54%) | 59 (8.22%) |  |
| Sleep quality |  |  |  | <0.001 |
| Very good | 891 (46.26%) | 611 (50.58%) | 280 (39.00%) |  |
| Good | 331 (17.19%) | 209 (17.30%) | 122 (16.99%) |  |
| Fair | 314 (16.30%) | 188 (15.56%) | 126 (17.55%) |  |
| Poor | 390 (20.25%) | 200 (16.56%) | 190 (26.46%) |  |
| Smoking Status |  |  |  | 0.055 |
| No | 1159 (60.18%) | 707 (58.53%) | 452 (62.95%) |  |
| Yes | 767 (39.82%) | 501 (41.47%) | 266 (37.05%) |  |
| Drinking Status |  |  |  | 0.036 |
| No | 1384 (71.86%) | 848 (70.20%) | 536 (74.65%) |  |
| Yes | 542 (28.14%) | 360 (29.80%) | 182 (25.35%) |  |
| Hypertension |  |  |  | 0.252 |
| No | 748 (38.84%) | 481 (39.82%) | 267 (37.19%) |  |
| Yes | 1178 (61.16%) | 727 (60.18%) | 451 (62.81%) |  |
| Diabetes |  |  |  | 0.846 |
| No | 1266 (65.73%) | 796 (65.89%) | 470 (65.46%) |  |
| Yes | 660 (34.27%) | 412 (34.11%) | 248 (34.54%) |  |
| Dyslipidemia |  |  |  | 0.824 |
| No | 1505 (78.14%) | 942 (77.98%) | 563 (78.41%) |  |
| Yes | 421 (21.86%) | 266 (22.02%) | 155 (21.59%) |  |
| Cancer |  |  |  | 0.707 |
| No | 1905 (98.91%) | 1194 (98.84%) | 711 (99.03%) |  |
| Yes | 21 (1.09%) | 14 (1.16%) | 7 (0.97%) |  |
| Lung disease |  |  |  | 0.599 |
| No | 1718 (89.20%) | 1081 (89.49%) | 637 (88.72%) |  |
| Yes | 208 (10.80%) | 127 (10.51%) | 81 (11.28%) |  |
| Psych-problem |  |  |  | 0.01 |
| No | 1904 (98.86%) | 1200 (99.34%) | 704 (98.05%) |  |
| Yes | 22 (1.14%) | 8 (0.66%) | 14 (1.95%) |  |
| Asthma |  |  |  | 0.496 |
| No | 1804 (93.67%) | 1135 (93.96%) | 669 (93.18%) |  |
| Yes | 122 (6.33%) | 73 (6.04%) | 49 (6.82%) |  |
| Liver disease |  |  |  | 0.058 |
| No | 1848 (95.95%) | 1167 (96.61%) | 681 (94.85%) |  |
| Yes | 78 (4.05%) | 41 (3.39%) | 37 (5.15%) |  |
| Heart disease |  |  |  | 0.163 |
| No | 1356 (70.40%) | 864 (71.52%) | 492 (68.52%) |  |
| Yes | 570 (29.60%) | 344 (28.48%) | 226 (31.48%) |  |
| Stroke |  |  |  | 0.133 |
| No | 1817 (94.34%) | 1147 (94.95%) | 670 (93.31%) |  |
| Yes | 109 (5.66%) | 61 (5.05%) | 48 (6.69%) |  |
| Kidney disease |  |  |  | 0.015 |
| No | 1773 (92.06%) | 1126 (93.21%) | 647 (90.11%) |  |
| Yes | 153 (7.94%) | 82 (6.79%) | 71 (9.89%) |  |
| Stomach or other digestive disease |  |  |  | 0.007 |
| No | 1489 (77.31%) | 958 (79.30%) | 531 (73.96%) |  |
| Yes | 437 (22.69%) | 250 (20.70%) | 187 (26.04%) |  |
| Memory-related disease |  |  |  | <0.001 |
| No | 1887 (97.98%) | 1194 (98.84%) | 693 (96.52%) |  |
| Yes | 39 (2.02%) | 14 (1.16%) | 25 (3.48%) |  |
| Arthritis or rheumatism |  |  |  | <0.001 |
| No | 1174 (60.96%) | 771 (63.82%) | 403 (56.13%) |  |
| Yes | 752 (39.04%) | 437 (36.18%) | 315 (43.87%) |  |
| ADL |  |  |  | <0.001 |
| Non-disability | 1568 (81.41%) | 1044 (86.42%) | 524 (72.98%) |  |
| Disability | 358 (18.59%) | 164 (13.58%) | 194 (27.02%) |  |
| IADL |  |  |  | <0.001 |
| Non-disability | 1517 (78.76%) | 1019 (84.35%) | 498 (69.36%) |  |
| Disability | 409 (21.24%) | 189 (15.65%) | 220 (30.64%) |  |
| Life satisfaction |  |  |  | <0.001 |
| Not at All Satisfied | 45 (2.34%) | 15 (1.24%) | 30 (4.18%) |  |
| Not Very Satisfied | 260 (13.50%) | 144 (11.92%) | 116 (16.16%) |  |
| Some What Satisfied | 1156 (60.02%) | 745 (61.67%) | 411 (57.24%) |  |
| Very Satisfied | 415 (21.55%) | 271 (22.43%) | 144 (20.06%) |  |
| Completely Satisfied | 50 (2.60%) | 33 (2.73%) | 17 (2.37%) |  |
| Health insurance |  |  |  | 0.838 |
| No | 110 (5.71%) | 70 (5.79%) | 40 (5.57%) |  |
| Yes | 1816 (94.29%) | 1138 (94.21%) | 678 (94.43%) |  |
| Hearing impairment |  |  |  | <0.001 |
| No | 1655 (85.93%) | 1098 (90.89%) | 557 (77.58%) |  |
| Yes | 271 (14.07%) | 110 (9.11%) | 161 (22.42%) |  |
| Pain |  |  |  | <0.001 |
| No | 1213 (62.98%) | 856 (70.86%) | 357 (49.72%) |  |
| Yes | 713 (37.02%) | 352 (29.14%) | 361 (50.28%) |  |
| Self-expectations of health status |  |  |  | <0.001 |
| Almost impossible | 189 (9.81%) | 94 (7.78%) | 95 (13.23%) |  |
| Not very likely | 445 (23.10%) | 226 (18.71%) | 219 (30.50%) |  |
| Maybe | 712 (36.97%) | 493 (40.81%) | 219 (30.50%) |  |
| Very likely | 279 (14.49%) | 191 (15.81%) | 88 (12.26%) |  |
| Almost certain | 301 (15.63%) | 204 (16.89%) | 97 (13.51%) |  |
| BMI category |  |  |  | 0.164 |
| Underweight | 81 (4.21%) | 57 (4.72%) | 24 (3.34%) |  |
| Normal weight | 1025 (53.22%) | 625 (51.74%) | 400 (55.71%) |  |
| Overweight | 671 (34.84%) | 425 (35.18%) | 246 (34.26%) |  |
| Obese | 149 (7.74%) | 101 (8.36%) | 48 (6.69%) |  |
| Depression |  |  |  | <0.001 |
| No | 1155 (59.97%) | 833 (68.96%) | 322 (44.85%) |  |
| Yes | 771 (40.03%) | 375 (31.04%) | 396 (55.15%) |  |
| Cataract surgery |  |  |  | 0.002 |
| No | 1870 (97.09%) | 1184 (98.01%) | 686 (95.54%) |  |
| Yes | 56 (2.91%) | 24 (1.99%) | 32 (4.46%) |  |
| Glaucoma |  |  |  | 0.01 |
| No | 1900 (98.65%) | 1198 (99.17%) | 702 (97.77%) |  |
| Yes | 26 (1.35%) | 10 (0.83%) | 16 (2.23%) |  |
| Age, Median (IQR) | 60.00 (54.00-68.00) | 59.00 (53.00-67.00) | 61.00 (56.00-68.00) | <0.001 |
| Chronic conditions, Median (IQR) | 2.00 (1.00-3.00) | 2.00 (1.00-3.00) | 2.00 (1.00-3.00) | <0.001 |
| Grip, Median (IQR) | 31.80 (25.13-39.00) | 33.00 (27.00-40.00) | 30.00 (24.00-36.42) | <0.001 |
| Cognition, Median (IQR) | 11.50 (9.00-13.50) | 12.00 (9.50-13.50) | 10.50 (8.00-13.00) | <0.001 |
| Waist, Median (IQR) | 88.25 (81.00-95.07) | 88.20 (81.00-95.53) | 88.40 (81.00-94.95) | 1 |
| WBC, Median (IQR) | 6.00 (5.00-7.30) | 6.00 (5.00-7.30) | 6.00 (5.00-7.40) | 0.908 |
| PLT, Median (IQR) | 205.00 (162.00-255.00) | 207.00 (161.75-257.00) | 202.00 (162.00-254.00) | 0.67 |
| HbA1c, Median (IQR) | 5.20 (4.90-5.60) | 5.20 (4.90-5.60) | 5.20 (4.90-5.60) | 0.362 |
| HB, Median (IQR) | 14.35 (13.20-15.70) | 14.50 (13.30-15.70) | 14.20 (13.10-15.67) | 0.047 |
| GLU, Median (IQR) | 108.00 (98.10-129.92) | 108.18 (98.46-130.32) | 107.28 (97.20-129.37) | 0.366 |
| TC, Median (IQR) | 194.07 (169.72-221.14) | 194.07 (169.72-221.23) | 194.27 (170.10-221.04) | 0.937 |
| TG, Median (IQR) | 121.25 (85.85-178.77) | 122.13 (86.73-184.08) | 117.71 (82.31-175.01) | 0.029 |
| HDLC, Median (IQR) | 46.01 (37.89-56.44) | 45.62 (37.89-55.28) | 47.17 (38.66-57.99) | 0.025 |
| LDLC, Median (IQR) | 116.75 (94.33-141.11) | 116.17 (93.94-140.34) | 116.95 (95.10-142.17) | 0.743 |
| CRP, Median (IQR) | 1.23 (0.65-2.55) | 1.25 (0.65-2.67) | 1.19 (0.64-2.34) | 0.348 |
| UA, Median (IQR) | 4.47 (3.70-5.41) | 4.51 (3.75-5.43) | 4.43 (3.68-5.33) | 0.055 |
| BUN, Median (IQR) | 15.27 (12.72-18.40) | 15.25 (12.74-18.29) | 15.27 (12.69-18.59) | 0.785 |
| CR, Median (IQR) | 0.78 (0.68-0.92) | 0.79 (0.68-0.93) | 0.77 (0.67-0.89) | 0.006 |
| CysC, Median (IQR) | 0.99 (0.87-1.15) | 0.99 (0.87-1.14) | 1.00 (0.88-1.15) | 0.235 |

*Note:* Median (interquartile range) was calculated for continuous variables, while frequencies and percentages were determined for categorical variables. The Wilcoxon rank-sum test was used to compare group differences for continuous variables, and Chi-squared tests were employed for categorical variables. ADL means Activities of Daily Living; IADL means Instrumental Activities of Daily Living; BMI means Body Mass Index; WBC means White blood cell; PLT means Platelets; HbA1c means Glycated hemoglobin; HB means Glycated Hemoglobin; GLU means Glucose; TC means Total Cholesterol; TG means Triglycerides; HDLC means High Density Lipoprotein-Cholesterol; LDLC means Low Density Lipoprotein Cholesterol; CRP means C-Reactive Protein. UA means Uric Acid; BUN means Blood Urea Nitrogen; CR means Creatinine; CysC means Cystatin C.

**Table S3 Comparison of differences in predictive factors between training and validation datasets in 2015.**

| **Variables** | **Training dataset** | **Validation dataset** | ***p-value*** |
| --- | --- | --- | --- |
|  | (n =2124) | (n =909) |  |
| Sex = male (%) | 1047 (49.3) | 470 (51.7) | 0.239 |
| marital = single (%) | 250 (11.8) | 111 (12.2) | 0.778 |
| Family residence = Urban (%) | 887 (41.8) | 384 (42.2) | 0.836 |
| Education level (%) |  |  | 0.954 |
| Elementary school | 552 (26.0) | 241 (26.5) |  |
| High school or above | 317 (14.9) | 129 (14.2) |  |
| Less than elementary school | 742 (34.9) | 321 (35.3) |  |
| Middle school | 513 (24.2) | 218 (24.0) |  |
| Sleeping time (%) |  |  | 0.478 |
| >8h | 195 (9.2) | 72 (7.9) |  |
| ≤6h | 1143 (53.8) | 488 (53.7) |  |
| 6–8h | 786 (37.0) | 349 (38.4) |  |
| Sleep quality (%) |  |  | 0.336 |
| 1 Very good | 1003 (47.2) | 447 (49.2) |  |
| 2 Good | 314 (14.8) | 131 (14.4) |  |
| 3 Poor | 370 (17.4) | 135 (14.9) |  |
| 4 Very poor | 437 (20.6) | 196 (21.6) |  |
| Smoking Status = Yes (%) | 1001 (47.1) | 432 (47.5) | 0.872 |
| Drinking Status = Yes (%) | 717 (33.8) | 343 (37.7) | 0.039 |
| Hypertension = Yes (%) | 1334 (62.8) | 555 (61.1) | 0.384 |
| Diabetes = Yes (%) | 678 (31.9) | 306 (33.7) | 0.37 |
| Dyslipidemia = Yes (%) | 789 (37.1) | 354 (38.9) | 0.371 |
| Cancer = Yes (%) | 43 (2.0) | 18 (2.0) | 1 |
| Lung disease = Yes (%) | 351 (16.5) | 165 (18.2) | 0.299 |
| Psych-problem = Yes (%) | 44 (2.1) | 20 (2.2) | 0.93 |
| Asthma = Yes (%) | 160 (7.5) | 68 (7.5) | 1 |
| Liver disease = Yes (%) | 181 (8.5) | 88 (9.7) | 0.337 |
| Heart disease = Yes (%) | 744 (35.0) | 328 (36.1) | 0.606 |
| Stroke = Yes (%) | 141 (6.6) | 49 (5.4) | 0.223 |
| Kidney disease = Yes (%) | 280 (13.2) | 138 (15.2) | 0.16 |
| Digestive disease = Yes (%) | 705 (33.2) | 320 (35.2) | 0.303 |
| Memory related disease = Yes (%) | 72 (3.4) | 26 (2.9) | 0.52 |
| Arthritis or rheumatism = Yes (%) | 1016 (47.8) | 429 (47.2) | 0.777 |
| ADL = Yes (%) | 515 (24.2) | 194 (21.3) | 0.092 |
| IADL = Yes (%) | 500 (23.5) | 214 (23.5) | 1 |
| Life satisfaction (%) |  |  | 0.594 |
| Completely Satisfied | 126 (5.9) | 63 (6.9) |  |
| Not at All Satisfied | 28 (1.3) | 15 (1.7) |  |
| Not Very Satisfied | 145 (6.8) | 52 (5.7) |  |
| Some What Satisfied | 1086 (51.1) | 468 (51.5) |  |
| Very Satisfied | 739 (34.8) | 311 (34.2) |  |
| Health insurance = Yes (%) | 1986 (93.5) | 847 (93.2) | 0.803 |
| Hearing impairment = Yes (%) | 326 (15.3) | 126 (13.9) | 0.318 |
| Pain = Yes (%) | 729 (34.3) | 308 (33.9) | 0.848 |
| Self-expectations of health status (%) |  |  | 0.227 |
| Almost certain | 285 (13.4) | 114 (12.5) |  |
| Almost impossible | 371 (17.5) | 137 (15.1) |  |
| Maybe | 777 (36.6) | 334 (36.7) |  |
| Not very likely | 449 (21.1) | 199 (21.9) |  |
| Very likely | 242 (11.4) | 125 (13.8) |  |
| BMI (%) |  |  | 0.29 |
| Normal weight | 91 (4.3) | 31 (3.4) |  |
| Obese | 1036 (48.8) | 471 (51.8) |  |
| Overweight | 825 (38.8) | 329 (36.2) |  |
| Underweight | 172 (8.1) | 78 (8.6) |  |
| Depression= Yes (%) | 793 (37.3) | 335 (36.9) | 0.833 |
| Cataract = Yes (%) | 58 (2.7) | 27 (3.0) | 0.806 |
| Glaucoma = Yes (%) | 32 (1.5) | 12 (1.3) | 0.82 |
| Age (mean (SD)) | 61.94 (8.35) | 61.54 (8.28) | 0.228 |
| Chronic conditions, (mean (SD)) | 2.94 (1.67) | 3.01 (1.71) | 0.333 |
| Grip, (mean (SD)) | 31.30 (9.39) | 31.98 (9.81) | 0.073 |
| Cognition, (mean (SD)) | 11.08 (3.36) | 11.01 (3.27) | 0.627 |
| Waist, (mean (SD)) | 88.05 (13.45) | 88.34 (11.94) | 0.579 |
| WBC, (mean (SD)) | 6.12 (1.83) | 6.14 (2.06) | 0.846 |
| PLT, (mean (SD)) | 207.10 (73.03) | 203.53 (82.00) | 0.234 |
| HbA1c, (mean (SD)) | 6.26 (1.27) | 6.24 (1.20) | 0.689 |
| HB, (mean (SD)) | 13.93 (1.94) | 13.95 (2.05) | 0.839 |
| GLU, (mean (SD)) | 110.26 (44.18) | 110.28 (46.14) | 0.991 |
| TC, (mean (SD)) | 186.88 (36.02) | 186.64 (37.90) | 0.867 |
| TG, (mean (SD)) | 154.16 (93.88) | 160.54 (103.13) | 0.096 |
| HDLC, (mean (SD)) | 50.14 (11.36) | 49.45 (11.10) | 0.124 |
| LDLC, (mean (SD)) | 104.14 (28.51) | 103.55 (30.34) | 0.61 |
| CRP, (mean (SD)) | 3.02 (6.51) | 3.05 (6.10) | 0.89 |
| UA, (mean (SD)) | 5.10 (1.42) | 5.19 (1.53) | 0.128 |
| BUN, (mean (SD)) | 15.67 (4.99) | 15.83 (4.71) | 0.411 |
| CR, (mean (SD)) | 0.82 (0.38) | 0.85 (0.41) | 0.107 |
| CysC, (mean (SD)) | 0.88 (0.28) | 0.89 (0.27) | 0.354 |

*Note:* Median (interquartile range) was calculated for continuous variables, while frequencies and percentages were determined for categorical variables. The Wilcoxon rank-sum test was used to compare group differences for continuous variables, and Chi-squared tests were employed for categorical variables. ADL means Activities of Daily Living; IADL means Instrumental Activities of Daily Living; BMI means Body Mass Index; WBC means White blood cell; PLT means Platelets; HbA1c means Glycated hemoglobin; HB means Glycated Hemoglobin; GLU means Glucose; TC means Total Cholesterol; TG means Triglycerides; HDLC means High Density Lipoprotein-Cholesterol; LDLC means Low Density Lipoprotein Cholesterol; CRP means C-Reactive Protein. UA means Uric Acid; BUN means Blood Urea Nitrogen; CR means Creatinine; CysC means Cystatin C.

**2. Details of Candidate Variables**

2.1 Sociodemographic characteristics include gender(0 = female, 1 = male), age（continuous）, marital status (0 = unmarried, 1 = married), family residence (0 = urban, 1 = rural), education qualification (1 = Less than elementary school, 2 = Elementary school, 3 = Middle school，4=High school or above), and health insurance (0 = no, 1 = yes) .

2.2 Lifestyle factors include average sleep duration（0=≤6h，1=6–8h，2=>8h）, sleep quality（0=Very good，1=Good，2=Fair，3=Poor）, smoking status（0 = no/quit, 1 = yes）, and alcohol consumption（0 = no/quit, 1 = yes）.

2.3 Health-related factors included pain experience（0 = no, 1 = yes）, life satisfaction（1=Not at all satisfied，2=Not very satisfied，3=Somewhat satisfied，4=Very satisfied，5=Completely satisfied）, self-expectations of future health status（1=Almost impossible，2=Not very likely，3=Maybe，4=Very likely，5=Almost certain）, cognition scores（continuous）, activities of daily living (ADL) (0=non-disability, 1=disability), instrumental activities of daily living (IADL) (0=non-disability, 1=disability).

2.4 Medical conditions consisted of self-reported diagnoses of hearing impairment（0 = no, 1 = yes）, hypertension（0 = no, 1 = yes）, dyslipidemia（0 = no, 1 = yes）, chronic lung disease（0 = no, 1 = yes）, liver disease（0 = no, 1 = yes）, heart disease（0 = no, 1 = yes）, stroke（0 = no, 1 = yes）, kidney disease（0 = no, 1 = yes）, asthma（0 = no, 1 = yes）, cancer（0 = no, 1 = yes）, digestive disorders（0 = no, 1 = yes）, memory-related illnesses（0 = no, 1 = yes）, arthritis or rheumatism（0 = no, 1 = yes）, depression（0 = no, 1 = yes）, Cataract（0 = no, 1 = yes）, Glaucoma（0 = no, 1 = yes）,and total number of chronic diseases（continuous）.

2.5 Objective measurements included grip strength, waist circumference,, and blood biomarkers such as white blood cell count(WBC), platelet count(PLT), fasting glucose(GLU), glycated hemoglobin (HbA1c), total cholesterol(TC), triglycerides(TG), high-density lipoprotein cholesterol (HDL-C), low-density lipoprotein cholesterol (LDL-C), serum creatinine, uric acid (UA), blood urea nitrogen (BUN), C-reactive protein (CRP) are all recorded as continuous, body mass index（0 = Underweight, 1 = Normal weight, 2= Overweight, 3 = Obese）.
